# DIET AND NUTRITION: Vitamin D Regulates MS Gene

**DOI:** 10.1289/ehp.117-a196a

**Published:** 2009-05

**Authors:** Carol Potera

Multiple sclerosis (MS), an autoimmune disease affecting some 2.5 million people worldwide, is thought to arise from a confluence of genetic and environmental factors. Dietary vitamin D intake has been associated with lower MS risk, and vitamin D deficiency has been associated with increased risk, but direct links between vitamin D and MS have not been identified. A team of Canadian and British researchers has now reported evidence that vitamin D interacts with a variant form of the *HLA-DRB1* gene, which has been associated with MS.

As reported in the 6 February 2009 issue of the online journal *PLoS Genetics*, study leader George Ebers, a clinical neurologist at the University of Oxford, United Kingdom, and colleagues examined cells with two copies of the HLA-DRB*15 form of *HLA-DRB1*. They identified a vitamin D response element (VDRE)—a short stretch of DNA that is a signature of genes regulated by vitamin D—next to the gene. When they examined DNA from study participants they found the same VDRE sequence in each of 322 individuals with two copies of HLA-DRB1*15 (including people with and without MS), but found different VDRE sequences in DNA samples from 168 study participants without HLA-DRB1*15. The researchers also showed that the VDRE sequence found in people with HLA-DRB1*15 could bind to the vitamin D receptor, and that the *HLA-DRB1* gene responded more strongly to vitamin D in cells with the HLA-DRB1*15 VDRE sequence than in cells without it.

“This is the first direct evidence that vitamin D regulates the gene,” says Ebers. He says that whereas the general public has a 1 in 1,000 chance of developing MS, the estimated risk of MS is 1 in 300 for people with one copy of the HLA-DRB1*1501 gene variant, and 1 in 100 for those with two copies of HLA-DRB1*1501.

The next step is determining how vitamin D and HLA-DRB1*15 might interact to modulate the autoimmune nature of MS. Epidemiologic and animal studies of MS point to involvement of the thymus gland early in development. The thymus produces T cells, a type of white blood cell involved in immune responses to foreign proteins (antigens). Each T cell is made with a unique antigen receptor that allows the immune system to react quickly to any foreign protein a person might encounter. However, T cells with antigen receptors that could respond to normal “self” proteins must be destroyed to prevent an autoimmune response. The researchers reason that a lack of vitamin D in the thymus in early life could limit this process, thus enabling “self-directed” T cells to survive and trigger an autoimmune response to the myelin sheath on nerve fibers—a classic feature of MS.

The authors propose that vitamin D supplementation at critical periods during pregnancy and early childhood might reduce the risk for developing MS—a view endorsed by Bruce Hollis, a nutritional biochemist and professor of pediatrics at the Medical University of South Carolina. Whereas the Institute of Medicine recommends 200 international units of vitamin D per day (IU/day) for people under age 50, including pregnant and lactating women and infants, Hollis wrote in the December 2007 issue of the *Journal of Bone and Mineral Research* that as much as 6,000 IU/day might be needed to maintain adequate blood levels of the vitamin in these populations. He is currently wrapping up NIH-funded research that suggests at least 2,000 IU/day may be an optimal intake. [For more information on vitamin D deficiency, see “Benefits of Sunlight: A Bright Spot for Human Health,” *EHP* 116:A160–A167 (2008)].

“Many obstetricians are oblivious to their patients’ vitamin D status and falsely assume that the miniscule amount of vitamin D contained in prenatal vitamins will meet the needs of their patients,” Hollis says. “In fact that amount is not even close.” Considering that the body makes up to 20,000 IU of vitamin D when exposed to sunlight for 30 minutes on a summer day, Ebers says 2,000 IU/day “is not a dangerous amount.”

